# Comprehensive Cis-Regulation Analysis of Genetic Variants in Human Lymphoblastoid Cell Lines

**DOI:** 10.3389/fgene.2019.00806

**Published:** 2019-09-10

**Authors:** Ying Wang, Bo He, Yuanyuan Zhao, Jill L. Reiter, Steven X. Chen, Edward Simpson, Weixing Feng, Yunlong Liu

**Affiliations:** ^1^Institute of Intelligent System and Bioinformatics, College of Automation, Harbin Engineering University, Harbin, Heilongjiang, China; ^2^Heilongjiang Provincial Hospital, Harbin, Heilongjiang, China; ^3^Center for Computational Biology and Bioinformatics, School of Medicine, Indiana University, Indianapolis, IN, United States; ^4^BioHealth Informatics, School of Informatics and Computing, Indiana University, Indianapolis, IN, United States

**Keywords:** functional genetic variants, quantitative trait loci (QTLs), genetic regulatory pattern, maximum likelihood estimation, independent regulation

## Abstract

Genetic variants can influence the expression of mRNA and protein. Genetic regulatory loci such as expression quantitative trait loci (eQTLs) and protein quantitative trait loci (pQTLs) exist in several species. However, it remains unclear how human genetic variants regulate mRNA and protein expression. Here, we characterized six mechanistic models for the genetic regulatory patterns of single-nucleotide polymorphisms (SNPs) and their actions on post-transcriptional expression. Data from Yoruba HapMap lymphoblastoid cell lines were analyzed to identify human cis-eQTLs and pQTLs, as well as protein-specific QTLs (psQTLs). Our results indicated that genetic regulatory loci primarily affected mRNA and protein abundance in patterns where the two were well-correlated. While this finding was observed in both humans and mice (57.5% and 70.3%, respectively), the genetic regulatory patterns differed between species, implying evolutionary differences. Mouse SNPs generally targeted changes in transcript expression (51%), whereas in humans, they largely regulated protein abundance, independent of transcription levels (55.9%). The latter independent function can be explained by psQTLs. Our analysis suggests that local functional genetic variants in the human genome mainly modulate protein abundance independent of mRNA levels through post-transcriptional mechanisms. These findings clarify the impact of genetic variation on phenotype, which is of particular relevance to disease risk and treatment response.

## Introduction

Single-nucleotide polymorphisms (SNPs) play an important role in the regulation of transcription and translation (Montgomery et al., 2010; Schafer et al., 2015). The central dogma states that DNA is transcribed into mRNA, which is then translated into protein. Within this simple model, SNPs can influence protein abundance through their effect on mRNA expression (Levine and Tjian, 2003; Goodrich and Kugel, 2010). However, genetic variants can also regulate protein abundance in a post-transcriptional way, regardless of transcription levels (Cox et al., 2007; White and Sharrocks, 2010; Foss et al., 2011; Battle et al., 2015). These mechanisms affect protein production and can be associated with complex traits or diseases. Moreover, genetic variants quantitatively affect the levels of transcripts and proteins in a manner that can be identified by mapping quantitative trait loci to transcript (eQTL) and/or protein (pQTL) abundance. Protein-specific QTLs (psQTLs) are genetic variants that affect protein abundance irrespective of changes in mRNA levels. Although such variants have been identified in mice (Chick et al., 2016), this global regulatory process has yet to be fully investigated in humans.

During the past decade, genome-wide association studies (GWAS) have identified thousands of regulatory genetic variants not only in humans but also in many other species, for varieties of complex traits ranging from disease to quantitative traits and including mRNA or protein levels (Stranger et al., 2005; Melzer et al., 2008; Ghazalpour et al., 2011; Majewski and Pastinen, 2011; Stark et al., 2014; Zhou et al., 2018d). Like many other molecular markers that have been discovered, these genetic variants can be utilized as potential diagnostic and therapeutic biomarkers in many cancer types (Zaenker and Ziman, 2013; Zhou et al., 2015b; Garrigos et al., 2018; Zhou et al., 2018a; Zhou et al., 2018b). However, GWAS have limitations: most focus primarily on detecting genetic variants associated with a single trait of interest, such as the expression of mRNA or protein, yet complex regulatory mechanisms are likely to affect protein levels. Recently, a study identified pQTLs at the proteome scale and statistically analyzed the multiple regulatory relationships existing between SNPs, mRNA, and protein. This suggested that local pQTLs were largely mediated through transcriptional mechanisms (Chick et al., 2016). However, these data derived from mice and were limited to pQTLs, and did not consider other potential regulatory variants such as eQTLs and psQTLs. Thus, a limited number of studies have considered the underlying genetic regulatory mechanisms found in humans.

In the present study, we assessed six regulatory relationship models in humans. The correlations between genotype, transcript levels, and protein abundance were quantified from lymphoblastoid cell lines (LCLs) of 62 unrelated HapMap Yoruba individuals from Ibadan, Nigeria (YRI). Our results show that genetic regulatory patterns in which transcription levels directly affected protein abundance were predominant in both humans and mice; however, one specific pattern was enriched in humans. Additionally, the regulatory loci underlying the human-enriched regulatory pattern were enriched in psQTLs that were predicted to independently affect protein abundance. This may be associated with a differential regulatory mechanism, with possible biological functional diversity between human and mice.

## Materials and Methods

### Datasets

Genome-wide genotypes and mRNA and protein quantification data from 62 YRI HapMap human LCLs were obtained from a recent study (Battle et al., 2015). We selected 4,340 genes in which mRNA and protein were quantified in at least half of the individuals for further analysis. Gene and protein expression data of 62 samples were downloaded from Supplementary Data 4 (Battle et al., 2015). Genotypes contained approximately 15.8 million variants imputed from either HapMap or the 1000 Genomes project (14.9M SNPs and 0.9M indels). SNPs and indels within a ±20-kb region of each gene and which had a minor allele frequency greater than 10% were selected as QTL candidates, leading to 2,118,301 variants. Although a ±20-kb region may be considered conservative, it is reasonable in our case because we are primarily interested in the difference between protein and RNA levels that can be explained by variants that function in cis. Corresponding genotype data can be obtained from: http://eqtl.uchicago.edu/dsQTL_data/GENOTYPES/.

We also downloaded local pQTLs from the study by Chick et al. (2016), which measured genome-wide transcript and protein expression in livers from 192 Diversity Outbred mice. For 6,707 proteins detected in at least half of the samples, the most probable models linking a QTL genotype to transcript and protein abundance were also obtained from the original paper. Here, we focus on the six models where a local QTL affects transcript or protein abundance, and obtained the number of local QTLs that can be best explained by each model from Table S6 (Chick et al., 2016).

### QTL Mapping

QTLs were identified in human YRI HapMap individuals using R software. Prior to QTL mapping analysis, we used standardization, quantile normalization, and principal components analysis to ensure that the data (mRNA and protein abundance) followed a standard normal distribution with no unidentified confounders (Battle et al., 2015).

In the first round of identification of regulatory patterns, we used only one pQTL for each gene corresponding to the smallest *p* value in linear regression, regardless of whether the *p* values were significant. In the second round, all eQTLs and pQTLs were mapped through linear regression analysis using the “lm” R package; psQTLs were identified using likelihood ratio test (LRT) with the following two linear models performed by the ”lrtest” function in “lmtest” R packages:

$$y=\beta_{0}+\beta_{1}x_{S}+\beta_{2}x_{R}+\varepsilon$$

$$y=\beta_{0}+\beta_{2}x_{R}+\varepsilon$$

where *x_S_* is the genotype, *x_R_* is the level of mRNA expression, and y is the level of protein expression. The *p* value was recorded as significant evidence. We filtered eQTLs, pQTLs, and psQTLs using a cutoff *p* value (4.8 × 10^−4^) that was determined at a false discovery rate (FDR) of 0.1 after multiple hypothesis corrections (Pickrell et al., 2010).

### Maximum Likelihood Model and Model Selection

For each candidate gene in the dataset, we evaluated six possible genetic regulatory relationships between SNP, and mRNA and protein abundance using the maximum likelihood model. The best model was selected by the minimum Bayesian information criterion (BIC) value. BIC values and corresponding weight values were calculated using the “bbmle” package of R.

It was assumed that the models of regulatory patterns were established based on a Markov chain. The maximum likelihood estimation for these models can be performed using joint probability distributions as follows:

(1) Pattern#1model:P(S,R,N)=P(S)P(R|S)P(N)

(2) Pattern#2model:P(S,R,N)=P(S)P(R)P(N|S)

(3) Pattern#3model:P(S,R,N)=P(S)P(R|S)P(N|S)

(4) Pattern#4model:P(S,R,N)=P(S)P(R|S)P(N|R)

(5) Pattern##5model:P(S,R,N)=P(S)P(R)P(N|S,R)

(6) Pattern#6model:P(S,R,N)=P(S)P(R|S)P(N|R,S)

where S is the SNP genotype, R is the mRNA level, and N is the protein level. P(R|S) and P(N|S) mean that the phenotype (mRNA and protein level) is associated with an SNP; P(N|R) in model #4 means that N is associated with R; and P(N|S, R) in model #5 means that N is associated with R, which may be affected by other SNPs or other common factors, but is unrelated to S. However, P(N|R, S) in model #6 means that N is associated with R, which may be influenced by other SNPs or other common factors as well as S.

It was assumed that mRNA and protein levels follow a normal distribution of N (0,1). We further assumed that traits R and N are normally distributed under each genotypic mean of a SNP, so that the likelihoods corresponding to each of the joint probability distributions were based on a normal probability density function.

(7) $$\text{Pattern}\#1\text{model}:\text{L}\left(\theta_{\text{1}}\right)=\prod_{l}^{m}\sum_{j=1}^{3}\text{p}\left({\text{S}}_{\text{j}}\right){\text{p(R}}_{\text{i}}{\text{|S}}_{\text{j}}{\text{)p(N}}_{\text{i}}\text{)}$$

(8) $$\text{Pattern}\#2\text{model}:\text{L}\left(\theta_{2}\right)=\prod_{l}^{m}\sum_{j=1}^{3}\text{p}\left({\text{S}}_{\text{j}}\right){\text{p(R}}_{\text{i}}{\text{)p(N}}_{\text{i}}{\text{|S}}_{\text{j}}\text{)}$$

(9) $$\text{Pattern}\#3\text{model}:\text{L}\left(\theta_{3}\right)=\prod_{l}^{m}\sum_{j=1}^{3}\text{p}\left({\text{S}}_{\text{j}}\right){\text{p(R}}_{\text{i}}{\text{|S}}_{\text{j}}{\text{)p(N}}_{\text{i}}{\text{|S}}_{\text{j}}\text{)}$$

(10) $$\text{Pattern}\#4\text{model}:\text{L}\left(\theta_{4}\right)=\prod_{l}^{m}\sum_{j=1}^{3}\text{p}\left({\text{S}}_{\text{j}}\right){\text{p(R}}_{\text{i}}{\text{|S}}_{\text{j}}{\text{)p(N}}_{\text{i}}{\text{|R}}_{\text{i}}\text{)}$$

(11) $$\text{Pattern}\#5\text{model}:\text{L}\left(\theta_{5}\right)=\prod_{l}^{m}\sum_{j=1}^{3}\text{p}\left({\text{S}}_{\text{j}}\right){\text{p(R}}_{\text{i}}{\text{)p(N}}_{\text{i}}{\text{|S}}_{\text{j}}{\text{,R}}_{\text{i}}\text{)}$$

(12) $$\text{Pattern}\#6\text{model}:\text{L}\left(\theta_{6}\right)=\prod_{l}^{m}\sum_{j=1}^{3}\text{p}\left({\text{S}}_{\text{j}}\right){\text{p(R}}_{\text{i}}{\text{|S}}_{\text{j}}{\text{)p(N}}_{\text{i}}{\text{|R}}_{\text{i}}{\text{,S}}_{\text{j}}\text{)}$$

where i is the individual from 1 to m, p(S_j_) is the probability of genotype S_j_ (j = 1, 2, 3) and was derived from the prior probability of the population, and p(R_i_) and p(N_i_) are from the normal probability density function. Other conditional probabilities were based on the normal conditional probability density function with means and variances for each component given by the following equations:

$$\text{p}\left({\text{R}}_{\text{i}}{\text{|S}}_{\text{j}}\right)\sim \text{N}\left(\mu =\mu_{{\text{RS}}_{\text{j}}},\sigma^{2}=\sigma_{\text{R}}^{2}\right)=\frac{1}{\sqrt{2\pi }\sigma }e^{-\frac{{\left(\text{x}-\mu \right)}^{2}}{2\sigma^{2}}}$$

$$\text{p}({\text{N}}_{\text{i}}|{\text{S}}_{\text{j}})\;\sim \text{N}\left(\mu =\mu_{{\text{NS}}_{\text{j}}},\sigma^{2}=\sigma_{\text{N}}^{2}\right)=\frac{1}{\sqrt{2\pi }\sigma }e^{-\frac{{\left(\text{x}-\mu \right)}^{2}}{2\sigma^{2}}}$$

$$\begin{array}{l} \text{p}({\text{N}}_{\text{i}}|{\text{R}}_{\text{i}})∼\text{N}\left(\mu =\mu_{\text{N}}+\rho \frac{\sigma_{\text{N}}}{\sigma_{\text{R}}}({\text{R}}_{\text{i}}-\mu_{\text{R}}),\sigma^{2}=(1-\rho^{2})\sigma_{\text{N}}^{2}\right)\\ \;=\frac{1}{\sqrt{2\pi }\sigma }e^{-\frac{{\left(\text{x}-\mu \right)}^{2}}{2\sigma^{2}}} \end{array}$$

$$\begin{array}{l} \text{p}\left({\text{N}}_{\text{i}}{\text{|S}}_{\text{j}},{\text{R}}_{\text{i}}\right)=\text{p}({\text{N}}_{\text{i}}|{\text{R}}_{\text{i}},{\text{S}}_{\text{j}})∼\\ \text{N}\left(\mu =\mu_{{\text{NS}}_{\text{j}}}+\rho \frac{\sigma_{\text{N}}}{\sigma_{\text{R}}}({\text{R}}_{\text{i}}-\mu_{\text{R}}),\sigma^{2}=(1-\rho^{2})\sigma_{\text{N}}^{2}\right)\;\\ =\frac{1}{\sqrt{2\pi }\sigma }e^{-\frac{{\left(\text{x}-\mu \right)}^{2}}{2\sigma^{2}}} \end{array}$$

where ρ is the correlation coefficient between R and N, and $\mu_{RS_{j}}$ and are $\mu_{{\text{NS}}_{\text{j}}}$ the genotype-specific means for R and N, respectively. The θ of each likelihood model was determined as follows:

$$\theta_{1}=\left(\mu_{{\text{RS}}_{\text{j}}},\sigma_{\text{R}}^{},\mu_{\text{N}},\sigma_{\text{N}}^{}\right),\text{j}=1,2,3,$$

$$\theta_{2}=\left(\mu_{\text{R}},\sigma_{\text{R}}^{},\mu_{{\text{NS}}_{\text{j}}},\sigma_{\text{N}}^{}\right),\text{j}=1,2,3,$$

$$\theta_{3}=\left(\mu_{{\text{RS}}_{\text{j}}},\sigma_{\text{R}}^{},\mu_{{\text{NS}}_{\text{j}}},\sigma_{\text{N}}^{}\right),\text{j}=1,2,3,$$

$$\theta_{4}=\left(\mu_{{\text{RS}}_{\text{j}}},\sigma_{\text{R}}^{},\mu_{\text{N}},\sigma_{\text{N}}^{},\rho ,\mu_{\text{R}}\right),\text{j}=1,2,3,$$

$$\theta_{5}=\left(\mu_{\text{R}},\sigma_{\text{R}}^{},\mu_{{\text{NS}}_{\text{j}}},\sigma_{\text{N}}^{},\rho \right),\text{j}=1,2,3,$$

$$\theta_{6}=\left(\mu_{{\text{RS}}_{\text{j}}},\mu_{\text{R}},\sigma_{\text{R}}^{},\mu_{{\text{NS}}_{\text{j}}},\sigma_{\text{N}}^{},\rho \right),\text{j}=1,2,3$$

We maximized the corresponding likelihood value for each model and evaluated the parameters using the maximum likelihood with initial values mean = 0 and standard deviation = 1. Then, the BIC value and the weight of each maximum likelihood model were calculated for each gene in the following functions using the “bbmle” package in R:

$${\text{BIC}}_{i}=-2\text{log}L_{i}+k_{i}\text{log}\left(n\right)$$

$${\text{wight}}_{i}=\frac{{\text{e}}^{-{\text{dBIC}}_{\text{i}}}}{\sum_{\text{k}=1}^{6}{\text{e}}^{-{\text{dBIC}}_{\text{k}}}}$$

where L_i_ is the maximum likelihood for the candidate model i, k_i_ is the number of parameters in the model i, and n is the sample size. wight_i_ is interpreted as the probability that model i is the best model, so Σ wight_i_ = 1. Lower values of BIC mean that the weight value was closer to 1. dBIC is the difference in BIC with respect to the BIC of the best candidate model: dBIC_i_ = BIC_i_ – min BIC. Larger values of dBIC mean that the weight value was closer to 0.

For each gene, we calculated BIC values and weights, which represented the relative quality and probability of six genetic regulatory models. The most likely model was predicted by the minimum BIC value and the maximum weight.

### Chromatin state enrichment analysis

Annotation of human LCL GM12878 chromatin states were obtained from: http://genome.ucsc.edu/cgi-bin/hgFileUi?db=hg19&g=wgEncodeBroadHmm (Accession: wgEncodeEH000784). In total, 15 chromatin states were annotated and used to segment the genome. We calculated the relative ratio showing whether a particular chromatin state of QTLs was enriched in the regulatory pattern by the formula ${\text{ratio}}_{ij}=\frac{\left(\#\;\mathrm{of}\;\mathrm{QT}L_{ij}\right)/\left(\#\;\mathrm{of}\;\mathrm{QT}L_{j}\right)}{\left(\#\;\mathrm{of}\;\mathrm{QT}L_{i}\right)/\left(\#\;\mathrm{of}\;\mathrm{QT}L\right)}$. Here, i is the chromatin state from 1 to 15, and j is the regulatory pattern from 1 to 6.

### Gene Functional Annotation

Gene functional enrichment analysis was performed by Database for Annotation, Visualization, and Integrated Discovery (DAVID) Bioinformatics Resources 6.8 (https://david.ncifcrf.gov/summary.jsp). The gene list of each regulatory pattern was submitted to run the functional annotation tool. The Benjamini method was chosen to perform multiple test correction. Modified Fisher’s exact *p* values were recorded for significantly enriched annotation terms.

## Results

### Regulatory Patterns With a Direct Effect From RNA to Protein Driven by Most Local Genetic Variants

**Figure 1:**
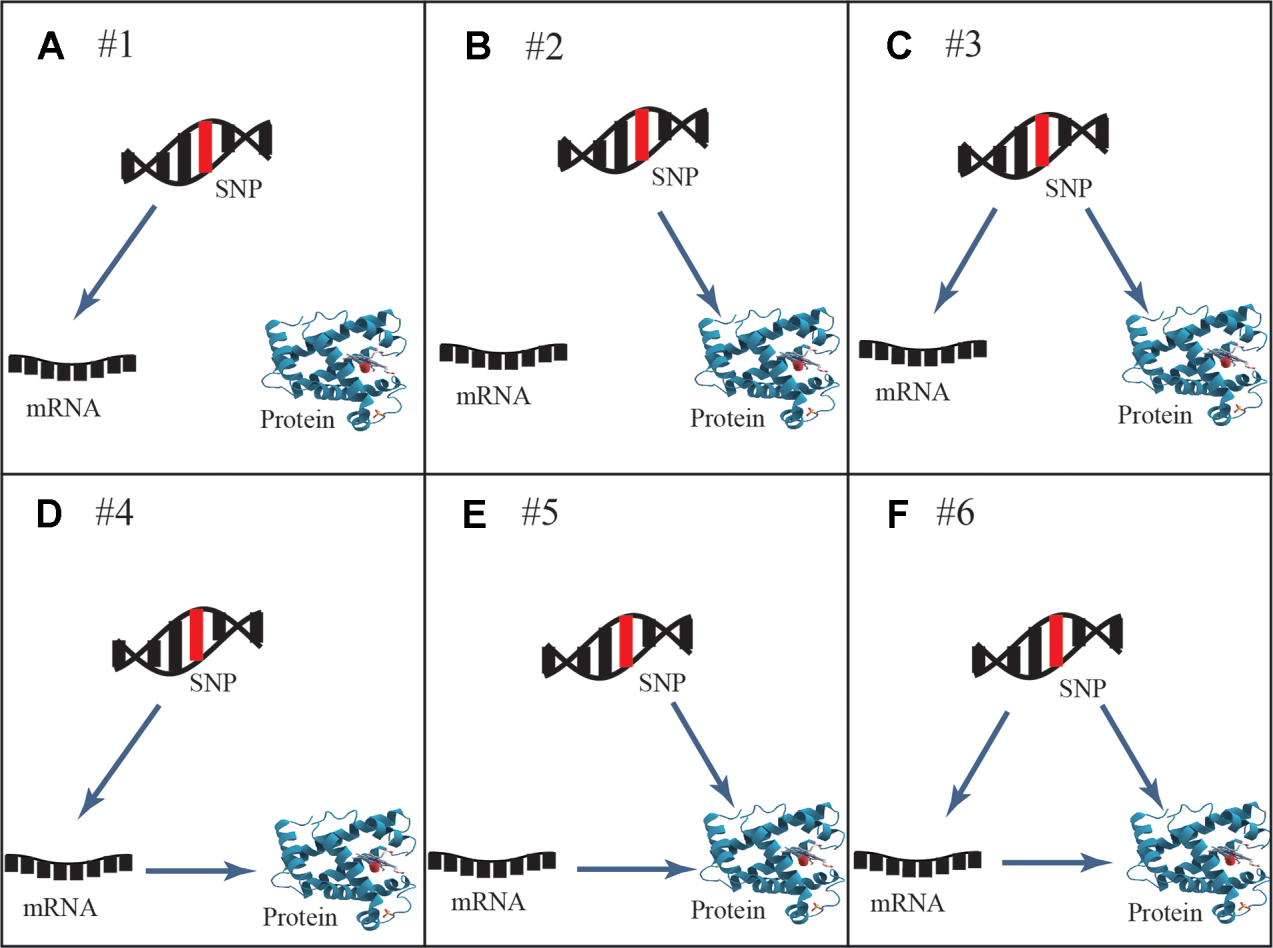
General genetic regulatory patterns across SNPs, mRNA, and protein. **(A, B)** Genetic variants known as eQTLs and pQTLs were considered to be the source of quantitative traits (mRNA or protein abundance), corresponding to pattern #1 and pattern #2. **(C)** Pattern #3 occurs when mRNA and protein share the same genetic variants while protein abundance is not associated with transcription levels; they are regulated by different independent mechanisms. **(D)** Pattern #4 occurs when genetic variants lead to the alteration of transcription, further to variation of protein abundance. **(E)** Pattern #5 occurs when genetic variants regulate protein abundance independently of mRNA levels. **(F)** Pattern #6 occurs when genetic variants and mRNA levels are dependent and co-regulate protein abundance. Overall, cis-acting SNPs act on mRNA and/or protein abundance through these likely regulatory patterns.

Six general patterns of how genetic variation leads to local regulation of transcript and/or protein abundance were investigated in human cells (**Figure 1**). Three regulatory patterns with SNPs that affect only mRNA or protein concentrations have previously been explored in multiple studies (Melzer et al., 2008; Pickrell et al., 2010; Ghazalpour et al., 2011; Majewski and Pastinen, 2011; Lourdusamy et al., 2012; Stranger et al., 2012; Wu et al., 2013; Hause et al., 2014; Consortium, 2015). These patterns also described how protein abundance was not determined by the levels of coding transcripts. The poor mRNA–protein correlation is supported by recent studies, which revealed influences from multiple processes including the spatial and temporal variations of mRNAs as well as the local availability of resources for protein biosynthesis (Liu et al., 2016). The three regulatory patterns are shown in the top row of **Figure 1**: SNPs that affect transcript levels without changing protein abundance (pattern #1, **Figure 1A**), SNPs that affect protein abundance without changing transcript levels (pattern #2, **Figure 1B**), and SNPs that affect transcript levels and protein abundance separately (pattern #3, **Figure 1C**). An additional three regulatory patterns that describe an association between mRNA and protein levels are shown in the bottom row of **Figure 1**: SNPs that affect transcript levels and thereby downstream protein abundance (pattern #4, **Figure 1D**), SNPs that play an independent role in regulating protein abundance, which is separately influenced by transcript levels (pattern #5, **Figure 1E**), and SNPs that cause both transcriptional and translational changes, but in which transcriptional changes also influence protein levels (pattern #6, **Figure 1F**).

To explore whether a predominant regulatory pattern exists in human cells, we selected 4,340 genes for which both mRNA and protein levels were measured in at least half of 62 unrelated HapMap Yoruba individuals for pQTLs identification. We only considered local SNPs (cis eQTLs, pQTLs and psQTLs) as candidate regulatory variants, which mapped to the target gene within a ±20-kb window. In our initial analysis, we used the same strategy published by Chick and colleagues (Chick et al., 2016) to select the candidate dataset that adopted only one pQTL for each gene with the lowest *p* value (regardless of whether the *p* value was significant). All pQTLs were identified by QTL mapping analysis based on a linear regression model. One of the six possible regulatory patterns between SNP, mRNA, and protein was determined for each pQTL using a maximum likelihood model and BIC scoring. The model with the smallest BIC value was considered to be the most probable regulatory relationship explained by the observed data.

**Figure 2:**
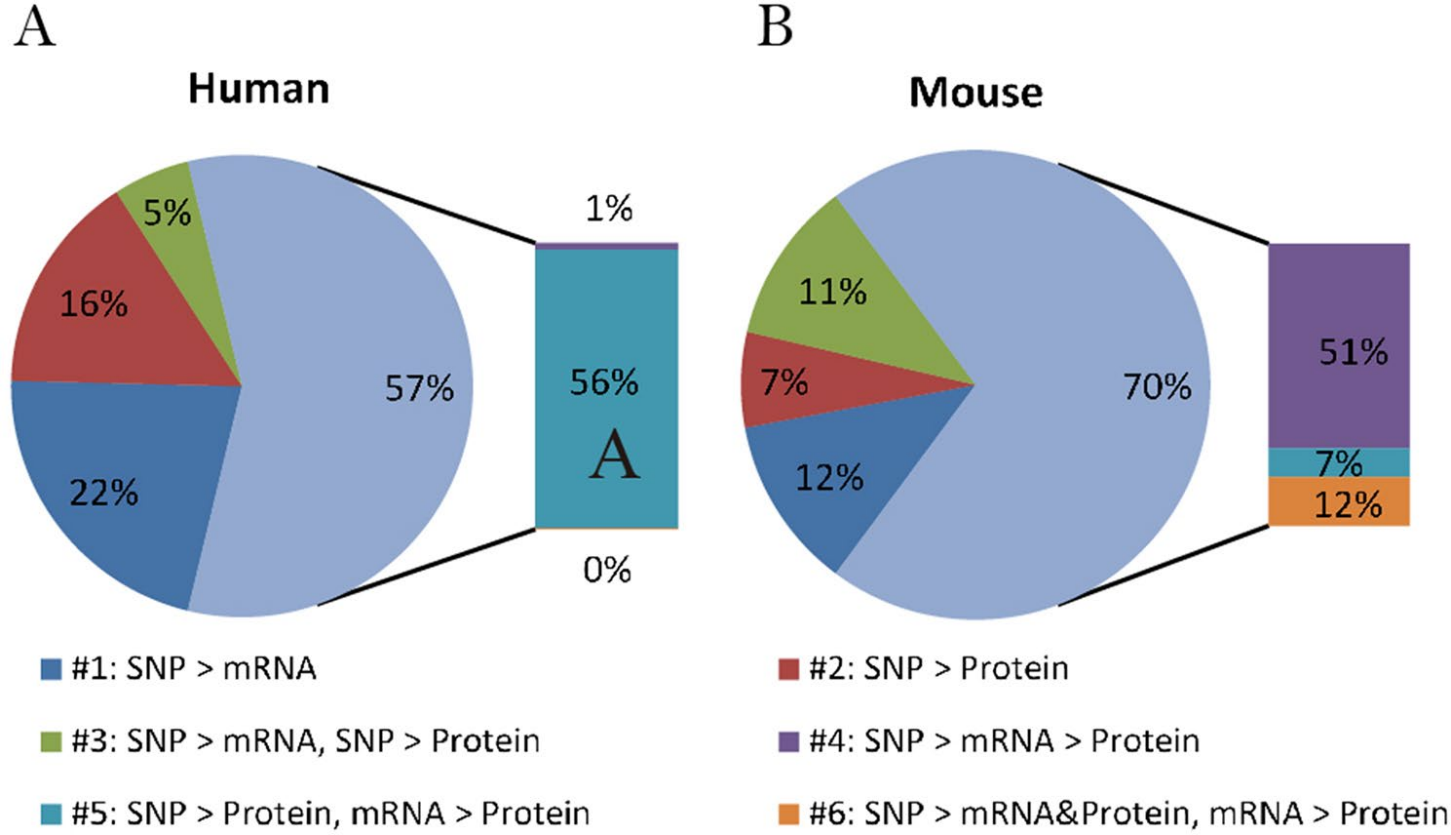
Distribution of genetic regulatory patterns. **(A)** Genetic regulatory patterns in human lymphoblastoid cell lines. **(B)** Local mouse regulation models according to Chick et al. (2016). This study identified the best local QTLs (±10 Mb of the gene midpoint) for each of the 6707 proteins in Diversity Outbred (DO) mice, and used Bayesian Information Criterion (BIC) to assess eight models between SNPs, mRNA, and protein. The six local regulation models associated with genetic variations were extracted to compare with those in humans.

By comparing the proportion of six models derived from human data in this study with those from mice summarized by Chick et al., we can better understand if there is a general relationship pattern through which local pQTLs affect protein expression, regardless of species. A similar distribution of regulatory patterns was observed in humans and mice (**Figure 2**). The predominant regulatory patterns in which mRNA and protein levels correlated well (patterns #4 to #6; **Figures 1D–F**) were observed both in humans (57.5%) and mice (70.3%). The Pearson correlation coefficients of mRNA and protein levels in patterns #4 to #6 were higher than that of patterns #1 to #3 (**Figure S1, A**). Almost half of the testing genes show a weak correlation with protein (Pearson correlation coefficient <0.2, *n* = 2554, **Figure S1, B**). Although the proportion of patterns #4 to #6 was predominant in both humans and mice, the pattern of genetic variants differed; in mice, local genetic variants primarily affected transcription levels (pattern #4, 51%), whereas in humans, they mainly regulated protein abundance regardless of the mRNA level (pattern #5, 55.9%). This indicates the existence of evolutionary differences in the mechanisms of genetic regulation and the fact that local human pQTLs preferentially regulate protein abundance through a post-transcriptional mechanism.

### QTL Analysis in Association With Regulatory Patterns

If protein abundance is regulated by pQTLs through a post-transcriptional mechanism, it would also correlate with transcriptional changes, so protein abundance may appear unchanged and potential regulatory pQTLs will not be found. To overcome this, we analyzed SNPs that were significantly associated with protein abundance while including the mediation of their coding mRNA levels, i.e., psQTLs. Given that protein abundance may be indirectly affected by eQTLs through changing mRNA levels, candidate datasets for model testing were extended to include all significant eQTLs, pQTLs, and psQTLs, rather than limiting them to the best pQTLs as above. Significant psQTLs were identified using the LRT; eQTLs and pQTLs were identified using QTL mapping analysis. All QTLs were filtered at an FDR threshold of 0.1. The regulatory patterns were then re-identified with extended QTL sets by the maximum likelihood estimation, where the aim was to find parameter values that made the observed data most likely to be in accordance with the statistical model. The best model was also determined by the minimum BIC score.

**Figure 3:**
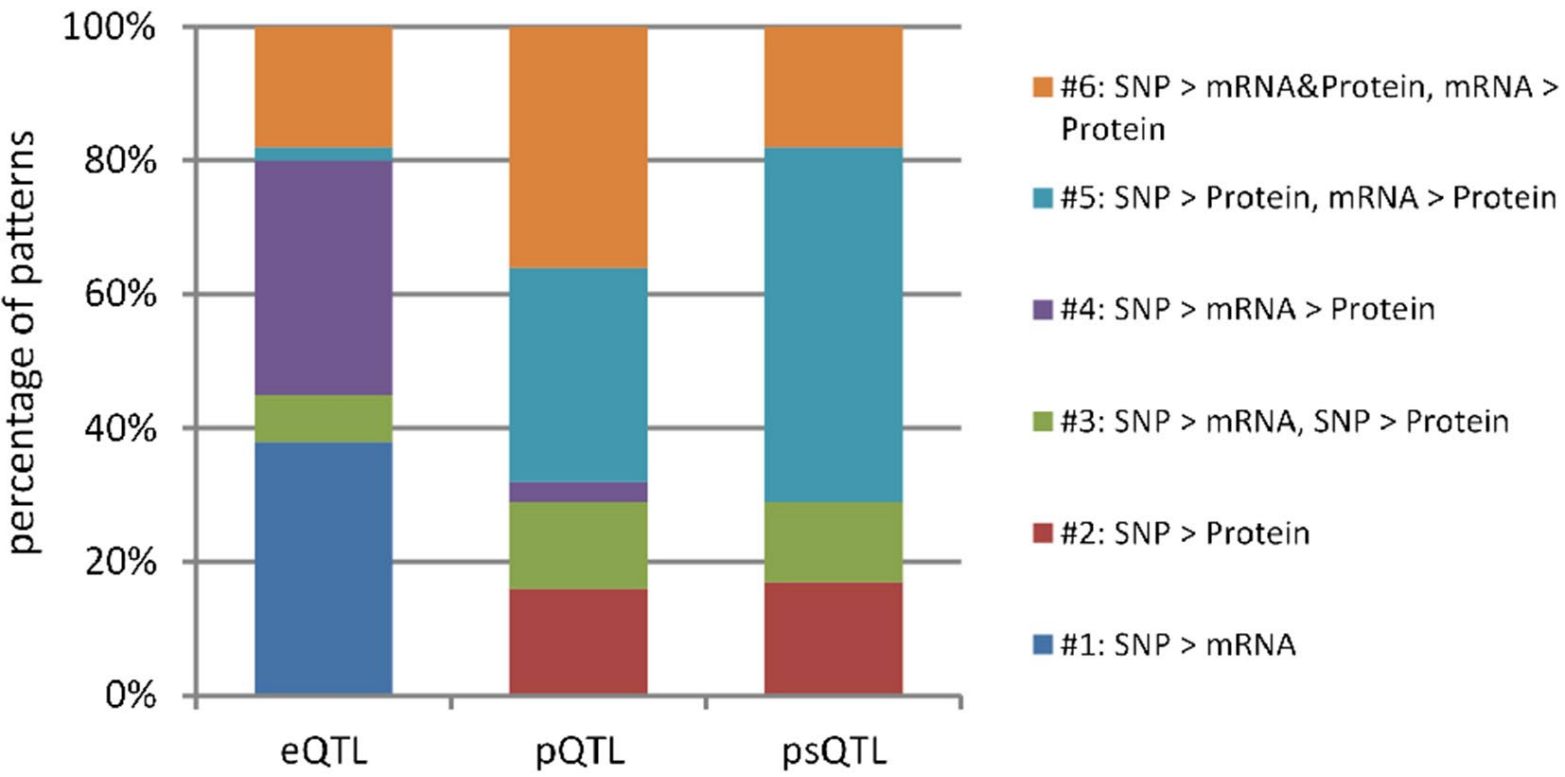
Genetic regulatory patterns in eQTLs, pQTLs, and psQTLs

In total, we identified 16,726 eQTLs, 8,364 pQTLs, and 5,475 psQTLs after multiple hypotheses correction (FDR = 0.1, *p* value = 4.8 × 10^−4^; **Table 1**) and obtained 23,241 combinations for all candidate QTLs and their associated mRNAs and proteins. Our results showed that pQTLs and psQTLs were specifically enriched in pattern #5 (SNP > protein, mRNA > protein), which indicated that many local human genetic variants affected protein abundance regardless of transcription levels. In contrast, most eQTLs that influenced protein abundance were found in pattern #4 (SNP > mRNA > protein, **Figure 3**). The same trend held true when a more stringent FDR of 0.01 was applied (**Figure S2**). This showed that eQTLs and pQTLs could affect protein abundance by different mechanisms. Although including a large number of eQTLs as input for model testing could change the overall proportion of patterns (**Figure S3**), the proportion of patterns including pQTLs and psQTLs has not been changed compared to the previous results (**Figure 2**, Human pQTLs; **Figure 3**, pQTLs and psQTL). Given that the proportion of pQTLs that overlapped with eQTLs is smaller in humans than in mice (**Figure S4**), humans have more genomic regulatory variants with independent functions than mice, which may explain the complex regulatory mechanism of species evolution.

**Table 1:** Number of cis-QTLs identified at FDR ≤ 0.1 and a cutoff *p* value ≤ 4.8 × 10^−^^4^.

QTL set	Individuals	Pairs^1^	cis-QTLs	qtlGenes^2^
eQTL	75	17,158	16,726	731
pQTL	62	8,438	8,364	440
psQTL	62	5,514	5,475	407

^1^Pairs were defined as combinations of SNPs and their associated mRNAs and proteins.

^2^qtlGenes were defined as genes with at least one QTL.

### Specificity of Cis-Regulatory QTLs in Variant Regulatory Patterns

To characterize cis-regulatory QTLs in the predominant regulatory patterns, we performed genomic location enrichment analysis based on the hypergeometric test. Human genome annotation was downloaded from the UCSC Genome Browser for specific regions, including promoter, gene body, upstream, and downstream regions. To examine the annotated functional elements of the identified genomic variants of different patterns, we performed an analysis of chromatin state enrichment using annotation of the human LCL GM12878. These states corresponded to active, weak, and inactive promoters, strong and weak enhancers, insulators, transcribed regions, and large-scale repressed and inactive domains (Ernst and Kellis, 2010; Ernst et al., 2011). The relative ratio shows whether a particular chromatin state of QTLs was enriched in the regulatory pattern (Materials and Methods). This analysis not only verified the identified QTL but also provided insights into the potential regulatory mechanisms underlying the different chromatin states.

**Figure 4:**
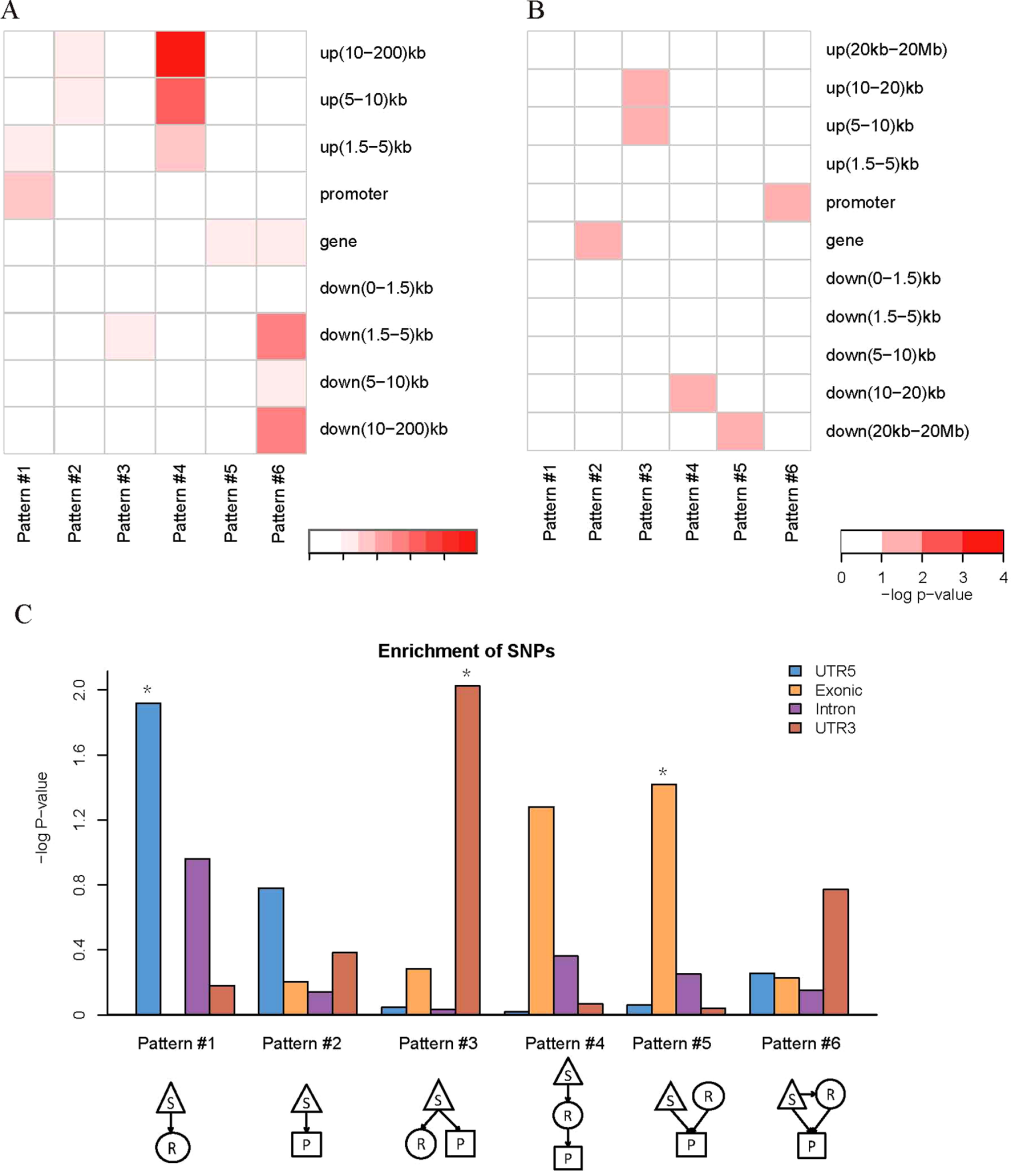
Location distributions of QTLs corresponding to regulatory patterns. **(A)** Genomic location enrichments of QTLs corresponding to regulatory patterns in humans. S, SNP; R, mRNA; P, protein. Color shading indicates the corrected *p* value ≤ 0.01 from hypergeometric tests. **(B)** Genomic location enrichments of QTLs corresponding to regulatory patterns in mice. Color shading indicates the corrected *p* value ≤ 0.1 from hypergeometric tests. **(C)** Enrichment of human regulatory patterns located in the gene body. The significance of enrichment is represented by the *p* value of hypergeometric tests. *p value ≤ 0.05

**Figure 5:**
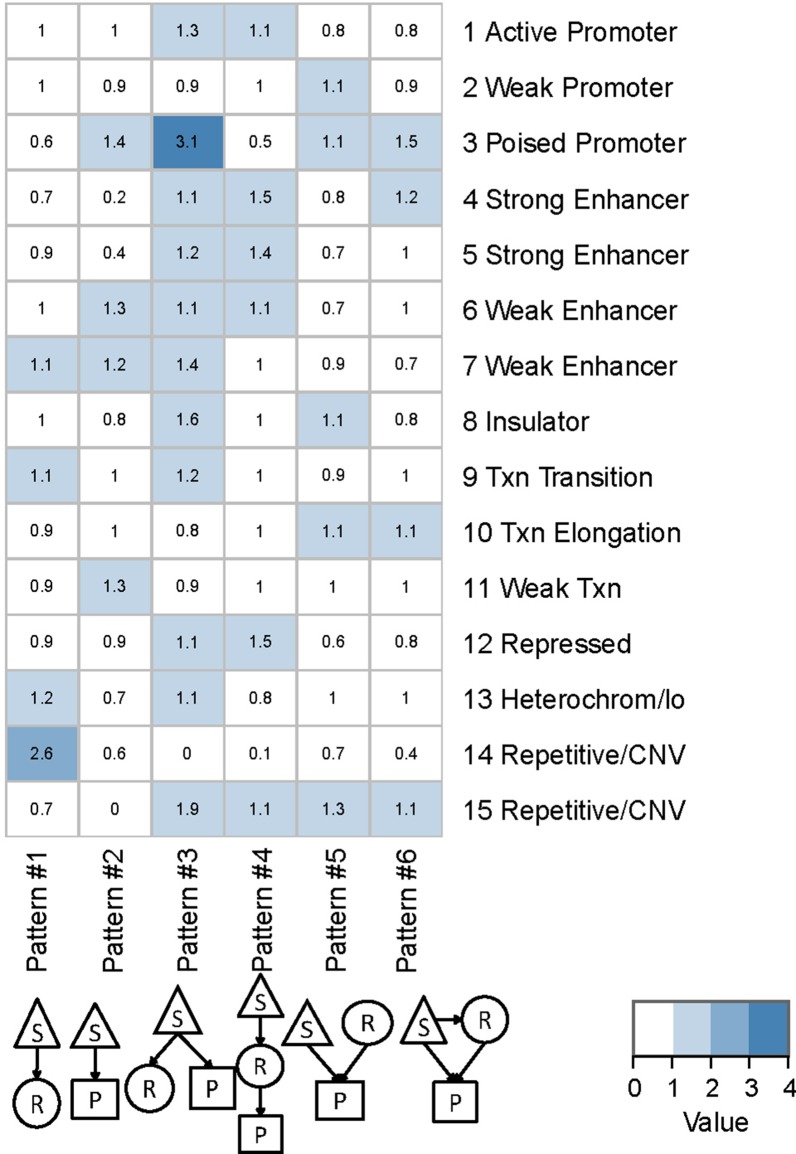
Chromatin states of QTLs corresponding to human regulatory patterns S, SNP; R, mRNA; P, protein. Color shading indicates the relative ratio, which is the proportion of particular state QTLs corresponding to each regulatory pattern divided by the ratio of that particular state QTL to the total QTL. The ratio value indicates whether a particular chromatin state of QTLs was enriched in each regulatory pattern, and is calculated by the following formula: ${\text{ratio}}_{ij}=\frac{\mathrm{NO}.\left(\mathrm{QT}L_{ij}\right)/\mathrm{NO}.\left(\mathrm{QT}L_{j}\right)}{\mathrm{NO}.\left(\mathrm{QT}L_{i}\right)/\mathrm{NO}.\left(\mathrm{QTL}\right)}$, where *i* is the chromatin state from 1 to 15 and *j* is the genetic regulatory pattern from 1 to 6.

We found that QTLs corresponding to different regulatory patterns tended to be enriched in different genomic regions (**Figure 4**). Because pattern #4 (SNP > mRNA > protein) and pattern #5 (SNP > protein, mRNA > protein) were the two main regulatory patterns across species, we focused further analysis on these patterns. Human QTLs showed many distinct features compared with those of mice (**Figures 4A, B**). QTLs corresponding to pattern #4 (SNP > mRNA > protein) were significantly enriched in the upstream regions of genes. These upstream regions were annotated to have chromatin states associated with active promoters, strong or weak enhancers, or polycomb repressed states (**Figure 5**). QTLs associated with pattern #5 were enriched in exon regions (**Figure 4C**). This indicates that the function of genomic variants depends on the genomic region. For example, regulatory variants located in promoter or enhancer regions tended to regulate gene expression through transcriptional mechanisms, while some regulatory variants located in the gene body independently regulated the protein abundance through post-transcriptional or translational mechanisms.

### Biological Functions Associated With Predominant Regulatory Patterns

**Figure 6:**
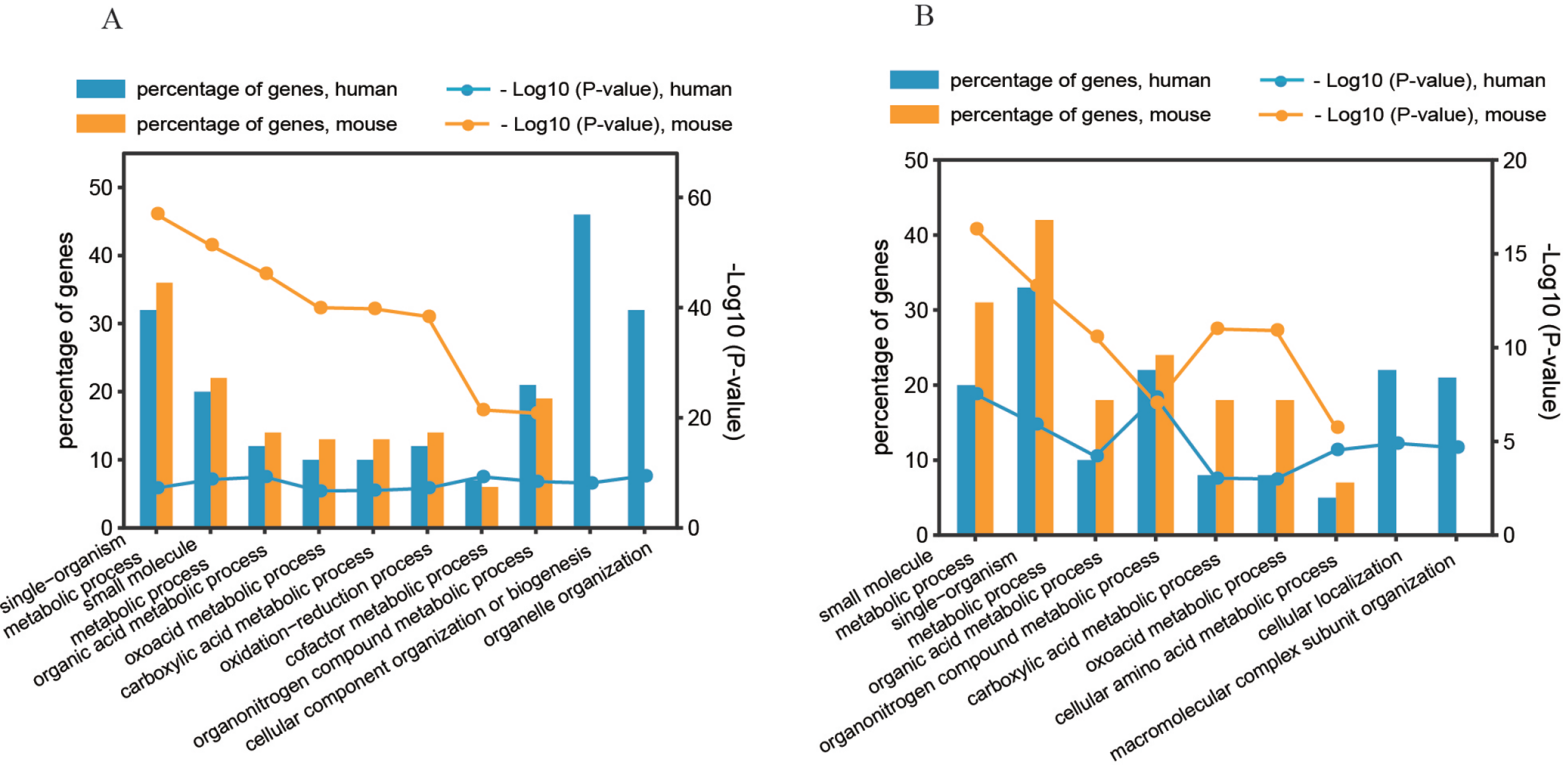
Gene annotation enrichment analysis of biological process. **(A)** Regulatory pattern #4: SNP > mRNA > Protein. **(B)** Regulatory pattern #5: SNP > Protein, mRNA > Protein.

To determine the significant biological functions of the genes affected by the two predominant regulatory patterns (pattern#4: SNP > mRNA > protein and pattern #5: SNP > protein, mRNA > protein), we analyzed the functional annotation enrichment according to Gene Ontology Biological Process terms using DAVID. Whole genome-wide genes were used as background for enrichment calculation. The significantly enriched terms of biological processes are shown in **Figure 6** and **Figure S5**. Genes in the two regulatory patterns were enriched in the basic biological processes of cellular activity, such as metabolic processes. However, some functions were differentially enriched. For example, human genes with regulatory pattern #4 were differentially enriched in cellular macromolecular complexes and organelle-related processes, including organelle organization, cellular component organization, and biogenesis (*p* value = 3.39 × 10^−10^ and 7.29 × 10^−9^, respectively, **Figure 6A**). Human genes with regulatory pattern #5 were specifically enriched in cellular localization and macromolecular complex subunit organization (*p* value = 1.26 × 10^−5^ and 2.19 × 10^−5^, respectively, **Figure 6B**). This suggested that when genetic variation acts as an independent regulator of protein abundance, genes are associated with cellular localization and macromolecular complex subunit organization. In general, our findings indicate the diversity of biological functions between human and mice, and the existence of differential genetic functions.

## Discussion

In this study, we examined human genetic variants that affect transcription levels and/or protein levels. Overall, our results show that human pQTLs near a gene primarily affect protein levels independently of transcription levels. These findings are supported by the fact that the mutant phenotype caused by the same genetic variants is also susceptible to gene expression (Vu et al., 2015), indicating that genetic variants and transcription levels together play a regulatory role.

We also investigated the different relationships between genetic variants and their associated mRNA and protein expression levels. We found that some genetic variants were best explained by regulatory patterns that do not correlate significantly with transcription and protein levels, as seen in patterns #1 to #3. Protein levels were not determined by transcription levels is the main difference between patterns #1 to #3 and #4 to #6. This suggested that there are potential regulatory networks with multiple genetic variants or other regulatory elements. It is well documented that protein levels are not always proportional to mRNA expression (De Sousa Abreu et al., 2009; Gry et al., 2009; Vogel and Marcotte, 2012) because regulation can occur at different levels, including RNA stability, translation efficiency, protein stability, and protein post-translational modifications (Zhou et al., 2015a; Zur et al., 2016; Zhou et al., 2017). Although our results indicate that protein levels were mainly controlled by local pQTLs following regulatory pattern #5 where SNPs and mRNA regulate protein levels independently, the actual regulatory network may not be limited by this. Thus, pattern #5 may be a sub-network in a complex regulatory network where mRNA and protein are affected by other SNPs or regulatory factors, such as *trans*-acting SNPs and/or co-regulated genes (Zhou et al., 2018c). The core gene, which has a direct effect on a change in the expected value of a phenotype, was found to be likely affected by large numbers of weak trans-acting (peripheral) variants through regulatory network and thus affect the trait indirectly (Liu et al., 2019). The overall effects on protein level are mediated through multiple cis and trans variants (and gene regulatory networks). Additionally, regulatory pattern #5 may involve the adjustment of elongation or termination phases of translation, which is consistent with the result of a recent study showing that some special amino acid sequences of nascent chain modulate polypeptide elongation speed in the ribosome (Chadani et al., 2017). The present study focused on how each individual SNP acts from RNA to protein in *cis*, and our results provide a complementary explanation for the regulatory control of protein levels.

A limitation of this study is that we only examined genetic variants in LCLs because of the need to collect three dimensions of data for the same individuals. LCL data are commonly used to investigate the role of regulatory variation in gene expression (Cheung et al., 2005; Duan et al., 2008; Dimas et al., 2009; Wu et al., 2013; Hause et al., 2014). Moreover, LCLs demonstrate a high level of replication across populations and samples (Li et al., 2008; Ding et al., 2010). Although gene expression was reported to show tissue specificity (Sonawane et al., 2017), many genomic variants regulate protein expression through post-transcriptional rather than transcriptional regulation, which provides additional explanation for the functional evolutionary difference among species. Hence, further investigation of the control of protein regulation across cell lines or tissues will be necessary for testing if specific regulatory patterns exist in tissues. Our study provides a suitable method that can be expanded for further application.

In summary, we found that protein abundance in human cells was primarily modulated by local QTLs and their coding transcripts. This was generally consistent with findings in mouse cells, although the predominant regulatory path of local pQTLs differed. Human functional variants play regulatory roles independent of transcription levels and can mainly be explained by psQTLs, implying that local genetic variants largely contribute to biological function through post-transcriptional mechanisms.

## Data Availability

Publicly available datasets were analyzed in this study. This data can be found here: http://eqtl.uchicago.edu/dsQTL_data/GENOTYPES/., http://genome.ucsc.edu/cgi-bin/hgFileUi?db=hg19&g=wgEncodeBroadHmm(Accession: wgEncodeEH000784),.

## Author Contributions

WF, YL, and YW designed the work program and drafted the manuscript. YW wrote the code and implemented the analysis. YW, BH, YZ, JR, SC, ES, WF, and YL participated in the writing of the paper and revising the manuscript. All authors read and approved the final manuscript.

## Funding

This research was funded by the National Natural Science Foundation of China (grant number 61471139), the Natural Science Foundation of Heilongjiang Province of China (grant number F2016006), and the HEU Fundamental Research Funds for the Central University (grant number 3072019CFG0401; HEUCFP201722).

## Conflict of Interest Statement

The authors declare that the research was conducted in the absence of any commercial or financial relationships that could be construed as a potential conflict of interest.

## Abbreviations

eQTL, expression quantitative trait loci; pQTL, protein quantitative trait loci; SNPs, single-nucleotide polymorphisms; psQTLs, protein-specific quantitative trait loci; LCLs, lymphoblastoid cell lines; GWAS, genome-wide association studies; YRI, Yoruba in Ibadan, Nigeria; BIC, Bayesian information criterion; LRT, likelihood ratio test; FDR, false discovery rate; GO, gene ontology; BP, biological process; DAVID, Database for Annotation, Visualization, and Integrated Discovery; MAF, minor allele frequency; PCA, principal components analysis.

## Supplementary Material

Supplementary Figure 1 Correlation between mRNA and protein levels. (A) Pearson correlation coefficient of mRNA and protein levels in three gene sets. Blue solid line represents genes that have patterns with weak or no correlation between mRNA and protein levels (patterns #1, 2, and 3); red solid line represents genes that have patterns with a strong correlation between mRNA and protein levels (patterns #4, 5 and 6); green dashed line represents the total gene set (n = 4340). Overlapping genes were removed from the two subsets in this plot. (B) The number of genes corresponding to different correlation coefficients (n = 4340).

Supplementary Figure 2 Distributions of genetic regulatory patterns in two subsets of QTLs. Genetic regulatory patterns have the same distribution characteristics in the two subsets of eQTL, pQTL, and psQTL (left, FDR = 0.01; right, FDR = 0.1).

Supplementary Figure 3 Overall distribution of genetic regulatory patterns including eQTLs, pQTLs, and psQTLs.

Supplementary Figure 4 Venn diagram of local QTL distribution for humans and mice. In humans, 38% of local pQTLs overlap with eQTLs; in mice (Chick JM, et al.), 80% of local pQTLs overlap with eQTLs.

Supplementary Figure 5 Functional annotation enrichment of Gene Ontology (GO) biological process (BP) terms. Gene sets were from regulatory pattern #4 (SNP > mRNA > protein) and pattern #5 (SNP > protein, mRNA > protein) of humans (pattern_H) and mice (pattern_M). Colors represent Fisher’s exact p value of a gene set enriched in a specific BP term. Dot size represents the gene percentage. The top 10 significant BP terms merged from each group are shown.
