# Supplementary figures and images for: Comprehensive Cis-Regulation Analysis of Genetic Variants in Human Lymphoblastoid Cell Lines
Source: Front Genet. 2019 Sep 10;10:806. doi: 10.3389/fgene.2019.00806 (PMC6747003; doi:10.3389/fgene.2019.00806)

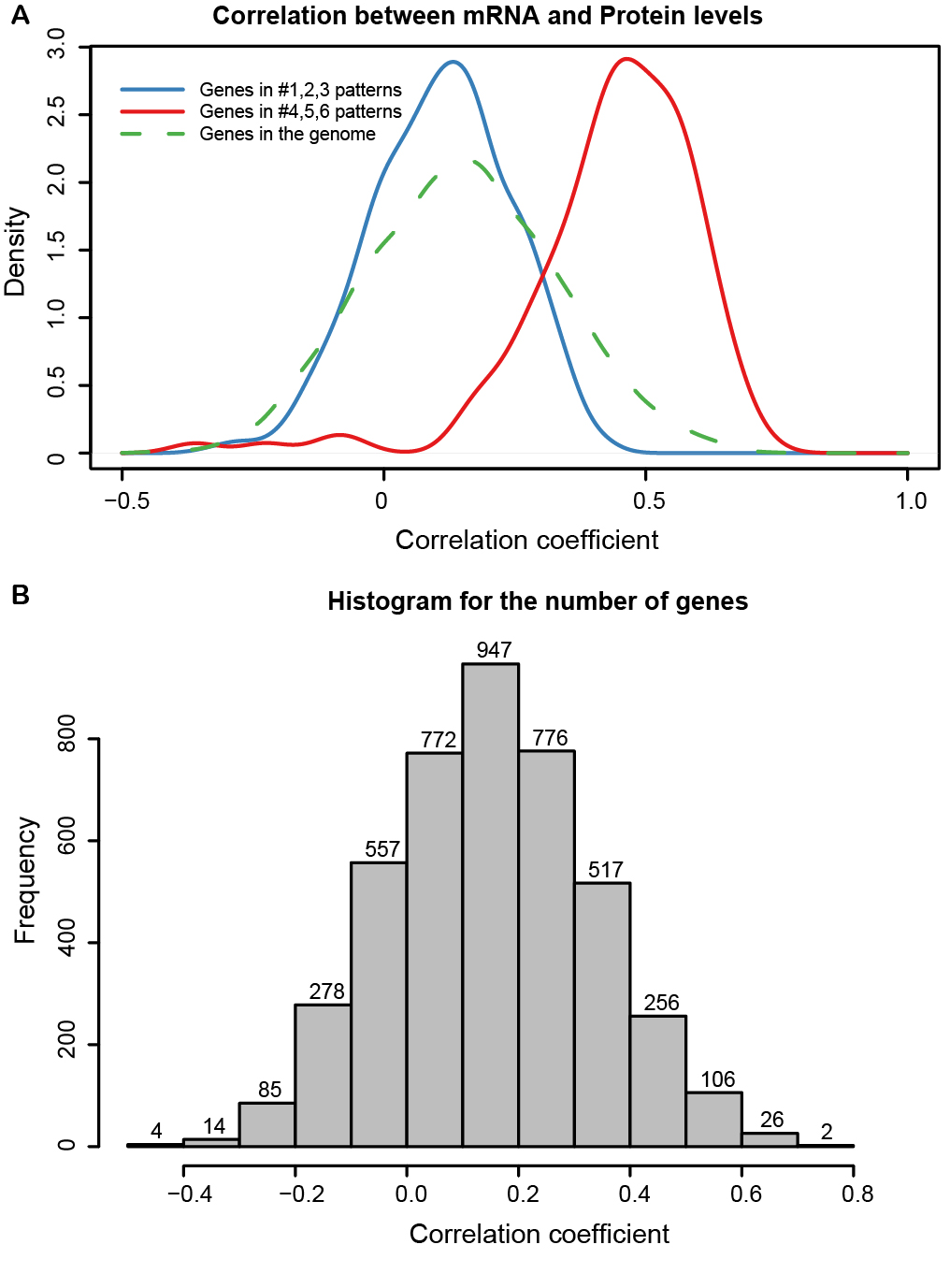

Supplement: Supplementary Figure 1 — Correlation between mRNA and protein levels. (A) Pearson correlation coefficient of mRNA and protein levels in three gene sets. Blue solid line represents genes that have patterns with weak or no correlation between mRNA and protein levels (patterns #1, 2, and 3); red solid line represents genes that have patterns with a strong correlation between mRNA and protein levels (patterns #4, 5 and 6); green dashed line represents the total gene set (n = 4340). Overlapping genes were removed from the two subsets in this plot. (B) The number of genes corresponding to different correlation coefficients (n = 4340). [file Image_1.jpeg]

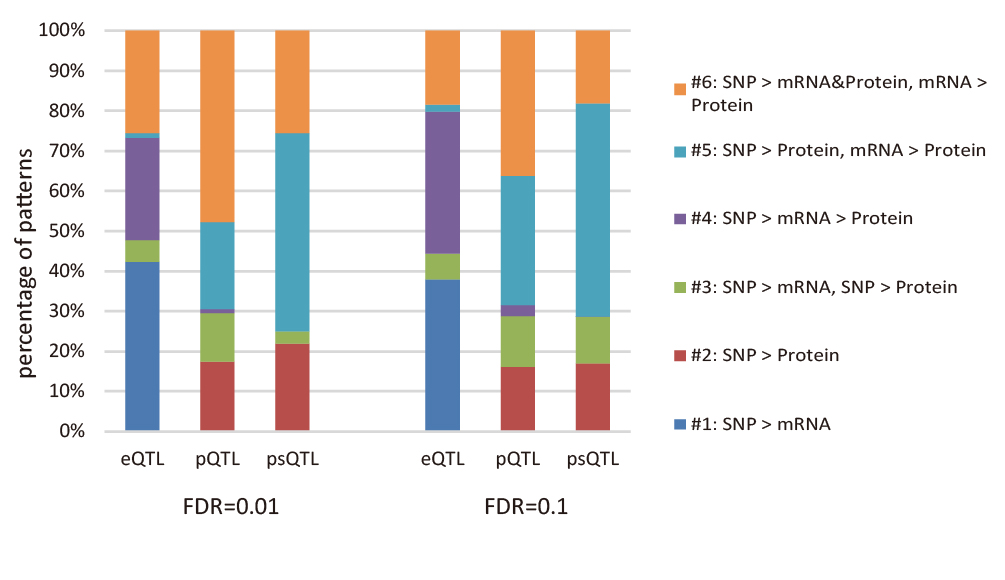

Supplement: Supplementary Figure 2 — Distributions of genetic regulatory patterns in two subsets of QTLs. Genetic regulatory patterns have the same distribution characteristics in the two subsets of eQTL, pQTL, and psQTL (left, FDR = 0.01; right, FDR = 0.1). [file Image_2.jpeg]

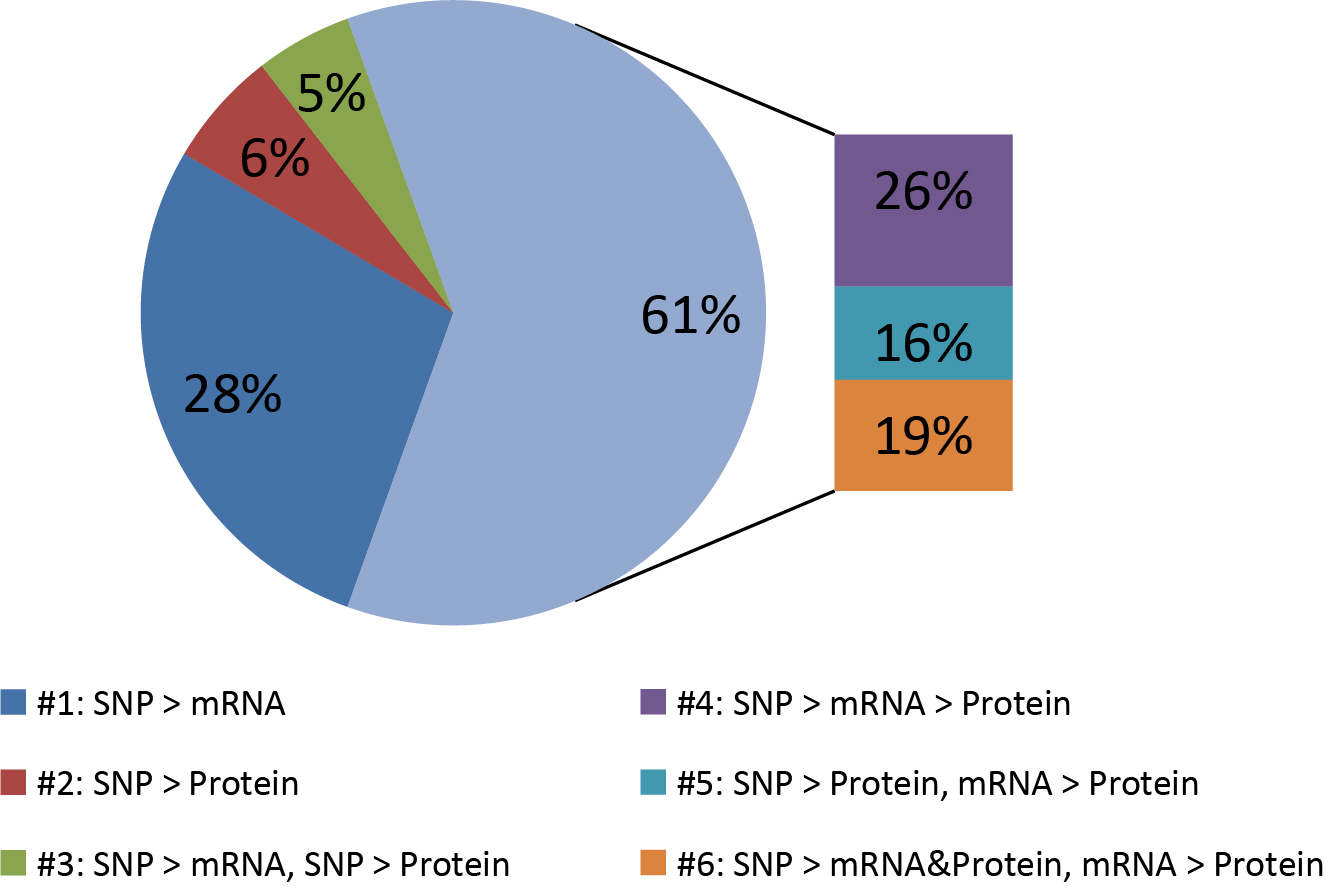

Supplement: Supplementary Figure 3 — Overall distribution of genetic regulatory patterns including eQTLs, pQTLs, and psQTLs. [file Image_3.jpeg]

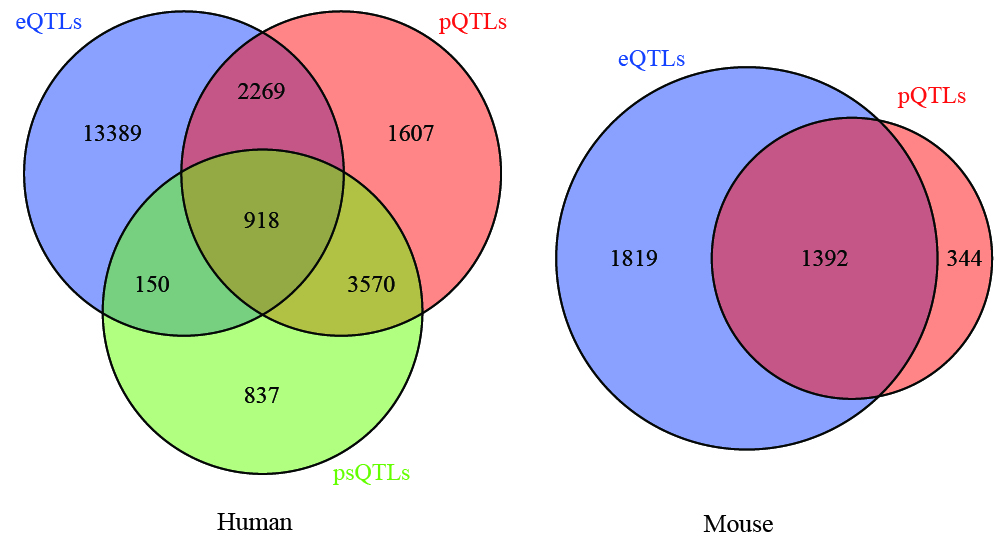

Supplement: Supplementary Figure 4 — Venn diagram of local QTL distribution for humans and mice. In humans, 38% of local pQTLs overlap with eQTLs; in mice (Chick JM, et al.), 80% of local pQTLs overlap with eQTLs. [file Image_4.jpeg]

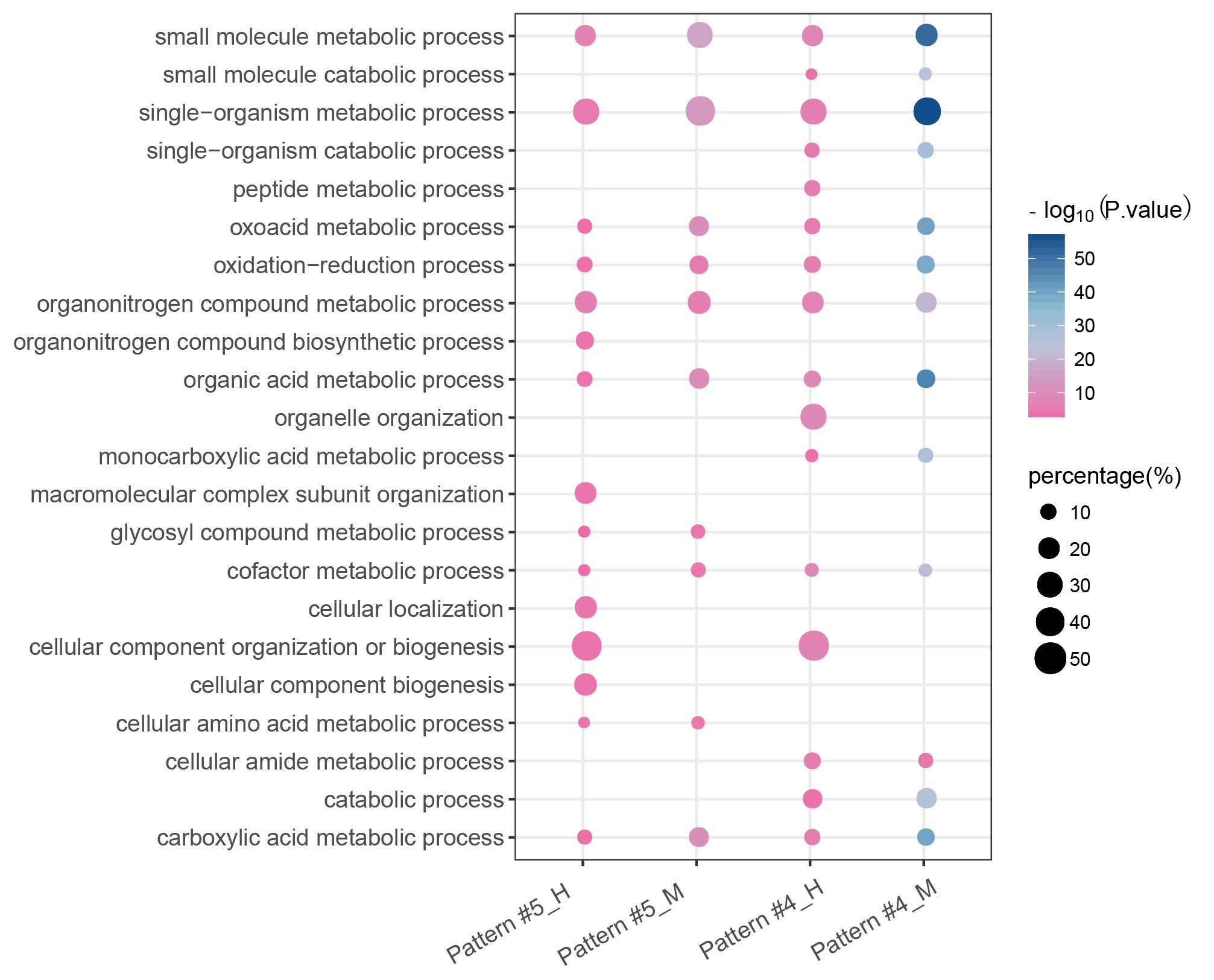

Supplement: Supplementary Figure 5 — Functional annotation enrichment of Gene Ontology (GO) biological process (BP) terms. Gene sets were from regulatory pattern #4 (SNP > mRNA > protein) and pattern #5 (SNP > protein, mRNA > protein) of humans (pattern_H) and mice (pattern_M). Colors represent Fisher’s exact p value of a gene set enriched in a specific BP term. Dot size represents the gene percentage. The top 10 significant BP terms merged from each group are shown. [file Image_5.jpeg]
